# Sheep farmers’ attitudes towards lameness control: Qualitative exploration of factors affecting adoption of the lameness Five-Point Plan

**DOI:** 10.1371/journal.pone.0246798

**Published:** 2021-02-09

**Authors:** Caroline M. Best, Alison Z. Pyatt, Janet Roden, Malgorzata Behnke, Kate Phillips

**Affiliations:** 1 Department of Veterinary Health & Animal Sciences, Harper Adams University, Newport, Shropshire, United Kingdom; 2 Equine Department, Hartpury University, Hartpury, Gloucestershire, United Kingdom; University of Lincoln, UNITED KINGDOM

## Abstract

In 2014, best-practice recommendations to treat and control lameness in sheep in the UK were consolidated into a national program, the Five-Point Plan (5PP). As recent evidence suggests that only the minority of sheep farmers are implementing all management practices listed in the 5PP, qualitative investigation is vital to ensure future promotion is aligned with psychological and contextual factors affecting farmers’ decision-making. This qualitative study sought to explore farmers’ attitudes and the factors affecting uptake of best-practice measures listed in the 5PP. Semi-structured interviews were conducted in 2019 with 12 sheep farmers from England and Wales. In accordance with Thematic Analysis and the principles of Grounded Theory, data collection and analysis were performed iteratively. Two overarching themes, delineated by subthemes, emerged during analysis; (1) Barriers to adoption of 5PP measures and (2) Motivation to adopt 5PP measures. Various farmer-centric factors and physical resources were identified as key barriers or obstacles that limited farmers’ ability to implement 5PP measures outright, or restricted their ability to make changes to facilitate future adoption. Conversely, internal and external influences were identified to increase farmers’ willingness and motivation to implement practices listed on the 5PP. Heterogeneity in farmer perceptions, attitudes, experiences and circumstances identified in this study highlights the difficulty in promoting a one-size-fits-all lameness control plan, where a unique combination of intrinsic factors, social influences, and physical restrictions affect implementation. Future initiatives should focus on removing barriers by changing farmers’ perceptions and mindset towards lameness control, and building farmers’ confidence in their ability to implement practices. Furthermore, farmers’ social licence to farm and their desire to improve their reputation within society, presents an important opportunity to further engage farmers in implementing control practices. Increasing peer-to-peer knowledge transfer opportunities and effective farmer-veterinarian communication and rapport could help establish 5PP measures as normative behaviours.

## Introduction

Lameness represents one of the most challenging health and welfare concerns in the UK sheep industry, with severe economic implications [1]. Over the last two decades, lameness of infectious origin has been the focus of extensive research; namely the treatment and control of footrot and contagious ovine digital dermatitis (CODD). Footrot (both clinical presentations; interdigital dermatitis and severe footrot) accounts for approximately 70% of lameness and is present in over 95% of flocks [2], whilst CODD is reported to affect between 35% and 60% of flocks [3, 4]. Most recent estimates of UK flock lameness prevalence suggest that on average 3.2% of ewes are lame [5].

In 2011, the Farm Animal Welfare Committee (FAWC) issued targets for farmers in Great Britain to reduce flock lameness to ≤5% by 2016 and ≤2% by 2021. As a result, in 2014, current best-practice recommendations were consolidated into the lameness Five-Point Plan (5PP), a practical, farm-level program of managements to help farmers reduce lameness in sheep. The 5PP was principally developed by the Food Animal Initiative (FAI) in conjunction with industry stakeholders, including levy boards and academic institutions. Five action points are listed in the 5PP which work in synergy to build resilience, reduce disease challenge and establish immunity; culling repeatedly lame sheep, treating individual clinical cases promptly and appropriately, quarantining incoming stock, avoiding the transmission of infection and vaccinating against footrot (Footvax®, MSD Animal Health Ltd). This national strategy has been widely promoted to UK sheep farmers predominantly through MSD Animal Health, levy boards, agricultural advisory services, veterinarians and farming press.

Although the efficacy of the 5PP has been documented in one case study of three flocks [6] and through a cross-sectional study of 532 UK sheep flocks [5], success of the 5PP ultimately depends on farmers’ willingness to adopt best-practice recommendations. In light of anecdotal industry concerns over 5PP uptake, one study reported only 5.8% of UK sheep farmers to adopt all five points of the 5PP [5]. Therefore, it is essential to understand farmers’ perceptions and attitudes towards implementation, considering many farmers will need to modify existing practices or farm facilities in order to adopt the 5PP.

The use of social science and the application of qualitative research philosophies has been key in the exploration of farmers’ perceptions of livestock disease control, from Johne’s disease [7, 8], mastitis [9, 10], and general disease biosecurity [11, 12] in dairy herds, to sheep parasite control [13, 14] and neonatal lamb health [15]. In addition, many qualitative studies have sought to explore the influence of personal factors (e.g., attitudes, perceptions, knowledge, experience, skills, opinions) and interpersonal factors (e.g., farmer-veterinarian relationships) on lameness treatment and control decisions in cattle [16–22]. Whilst this literature provides insight into cattle farmers’ perceptions of lameness control, there are few equivalent studies in the UK sheep industry. As a result of inherent differences between cattle and sheep farming systems, in addition to differences in the aetiology and pathogenesis of ovine and bovine lameness, directly extrapolating farmers’ perceptions of lameness control in the cattle industry to the sheep industry would be inappropriate.

Nonetheless, existing published studies have been valuable in our understanding of sheep farmers’ perceptions of lameness and its control. In a 2013 questionnaire study, negative emotions towards lameness, perceived production cycle limitations and insufficient knowledge of footrot transmission were identified as fundamental barriers leading to delayed treatment of lame sheep with footrot [23]. Lack of facilities and logistical constraints were also identified as barriers to treating lame sheep [24], although this was not the main focus of this study and not explored in detail. The barriers, mechanisms and motivators to adopting the “six steps to sound sheep” framework [25] was also investigated in a subsequent study [26]. Here, economics, alongside constraints on practicality, time and habit were identified to be among key barriers to changing lameness management practices, although ironically, saving time and money were also perceived as benefits of implementing control measures leading to sustained change. Tacit knowledge, derived from working alongside trusted farmers, was also reported to dominate agricultural students’ beliefs in treating lame sheep [27]. Here, some young farmers continued to practice foot trimming, despite being taught best-practice at agricultural college.

Whilst previous studies have explored the factors affecting uptake of some individual best-practice measures, no published studies have explored farmers’ attitudes towards adopting the five measures listed on the 5PP, nor the implementation of the 5PP in its entirety. As a national lameness control program, understanding the factors affecting implementation is integral to driving forward best-practice adoption and subsequent lameness reduction. The importance of understanding barriers has been previously highlighted, in order to inform future initiatives to encourage best-practice adoption [28]. By exploring if, how and why sheep farmers adopt 5PP management practices, future messages could be actively framed around factors that motivate farmers to implement sustained, long-term changes. To fill this gap in our understanding, the aim of this study was to recognise sheep farmers’ attitudes towards the 5PP, to explore the factors affecting its implementation. Our study builds upon previous research [5], in providing qualitative explanation to the low uptake of the 5PP in its entirety.

## Materials and methods

This study was approved by the Ethics Committee at Harper Adams University, Shropshire, UK. Sheep farmers previously engaged in a survey on sheep lameness management [5], who provided consent to be contacted, were invited for interview. Farmers were provided with an information sheet outlining the purpose and aims of the study, and the intention the study would contribute towards doctoral research.

### Data collection

Qualitative research methodologies of thematic analysis, and the principles of Grounded Theory were selected as techniques to yield rich and detailed data [29] in the exploration of farmer attitudes and experiences toward the 5PP. Qualitative methodologies have been widely used in studies exploring farmer decision-making and attitudes towards farm management and disease control [11, 30–32]. Purposive heterogenous sampling of participants was utilised to reflect the variation in sheep farmers, such as demographical variation and enterprise size, and to ensure germane contributions.

Semi-structured interviews were used in this study to explore farmers’ experiences, opinions and attitudes in their own words [33]. An interview guide (S1 File) was devised to standardise the interview process and to cover key concepts, concurrently allowing participants to expand upon issues important to them. A funnel-shaped questioning approach was adopted [33]; interviews commenced with generic questions relating to the farmer, farm and flock, and progressed to specific questions concerning attitudes, perceptions and experiences of the 5PP. Introductory questions helped to put the farmer at ease and build rapport [34]. Farmers were encouraged to respond freely throughout the interview. Questions were developed by all authors based on extant literature, industry experience, and following reflection on the data described in the previous study [5]. A pilot study and subsequent detailed feedback enabled appropriate modification prior to commencement of interviews.

Interviews were conducted by the primary researcher (CMB), between June and September 2019. CMB received training in a course on qualitative interview methodologies. Data saturation was achieved after interviewing 11 farmers, when no new themes emerged from the data collected [35]. Completion of an additional interview, 12, confirmed this decision. Four interviews were conducted face-to-face at the farmers’ location of choice, whilst eight interviews were conducted by telephone.

The final, heterogeneous sample of farmers (Table 1) was considered to be illustrative of sheep farmers in England and Wales, but not necessarily representative. Eight farmers had previously attended an agricultural college or university. All farmers interviewed were responsible, or jointly responsible, for making flock management decisions. All flocks had a history of footrot, but not all had experienced CODD. On average, farmer-estimated flock percentage lameness at the time of interview was 3.3%, ranging from 1% to 10%.

**Table 1:** Summary of participating sheep farmers (*n* = 12) from England and Wales, by farmer age, flock size, enterprises on farm and method of interview.

Farmer ^a^	Age ^b^	Flock size ^c^	Enterprises	Farm location	Method
1	25–35	>1000	Sheep only	South East	Telephone
2	25–35	251–500	Sheep and beef cattle	East Midlands	Telephone
3	46–55	501–1000	Sheep only	South West	Telephone
4	56–65	501–1000	Sheep and beef cattle	South West	Telephone
5	56–65	501–1000	Sheep and arable	West Midlands	Face-to-face
6	36–45	501–1000	Sheep and beef cattle	South West	Telephone
7	46–55	50–100	Sheep only	North West	Telephone
8	25–36	501–1000	Sheep and beef cattle	Wales	Telephone
9	25–36	251–500	Sheep and dairy cattle	South West	Face-to-face
10	36–45	251–500	Sheep only	West Midlands	Face-to-face
11	56–65	50–100	Sheep only	Wales	Telephone
12	46–55	101–250	Sheep and beef cattle	Wales	Face-to-face

^a^ Unique number identifier.

^b^ Years (category).

^c^ Number of ewes (category).

Interviews averaged 44 minutes in length (range: 21–78 minutes). All interviews were audio-recorded using a SONY IC Recorder. Digital audio files (MP3) were saved and stored in line with requirements of the General Data Protection Regulation (GDPR) and the UK Data Protection Act.

### Data analysis

Interviews were transcribed *verbatim* by CMB, and farmers were numbered to maintain confidentiality. Qualitative analysis was performed by CMB using specialist Qualitative Data Analysis (QDA) software (NVivo v.12, QSR International Pty Ltd, Australia), with support from an experienced qualitative researcher with advanced level NVivo training (AZP). In line with Thematic Analysis and the principles of Grounded Theory, data collection and analysis were performed iteratively. Transcripts were analysed using constant comparison techniques and followed the six key stages of Thematic Analysis [29]; (1) transcripts were read and re-read repeatedly to enable familiarisation with the content prior to commencement of coding; (2) coding was conducted to generate concise labels for important features; (3) coherent and meaningful patterns in the codes were identified forming themes; (4) themes were reviewed to ensure that they reflected the data; (5) themes were defined and named with appropriate detailed analysis; (6) themes were contextualised against existing literature and supporting evidence. Example quotes were used to provide further detail to qualitative themes identified around all aspects of the 5PP. As UK farmers refer to veterinarians as ‘vets’, all quotes were amended to ‘veterinarian’ to suit an international audience. A thematic map was created to visually depict inter-relationships between themes.

## Results

**Fig 1:**
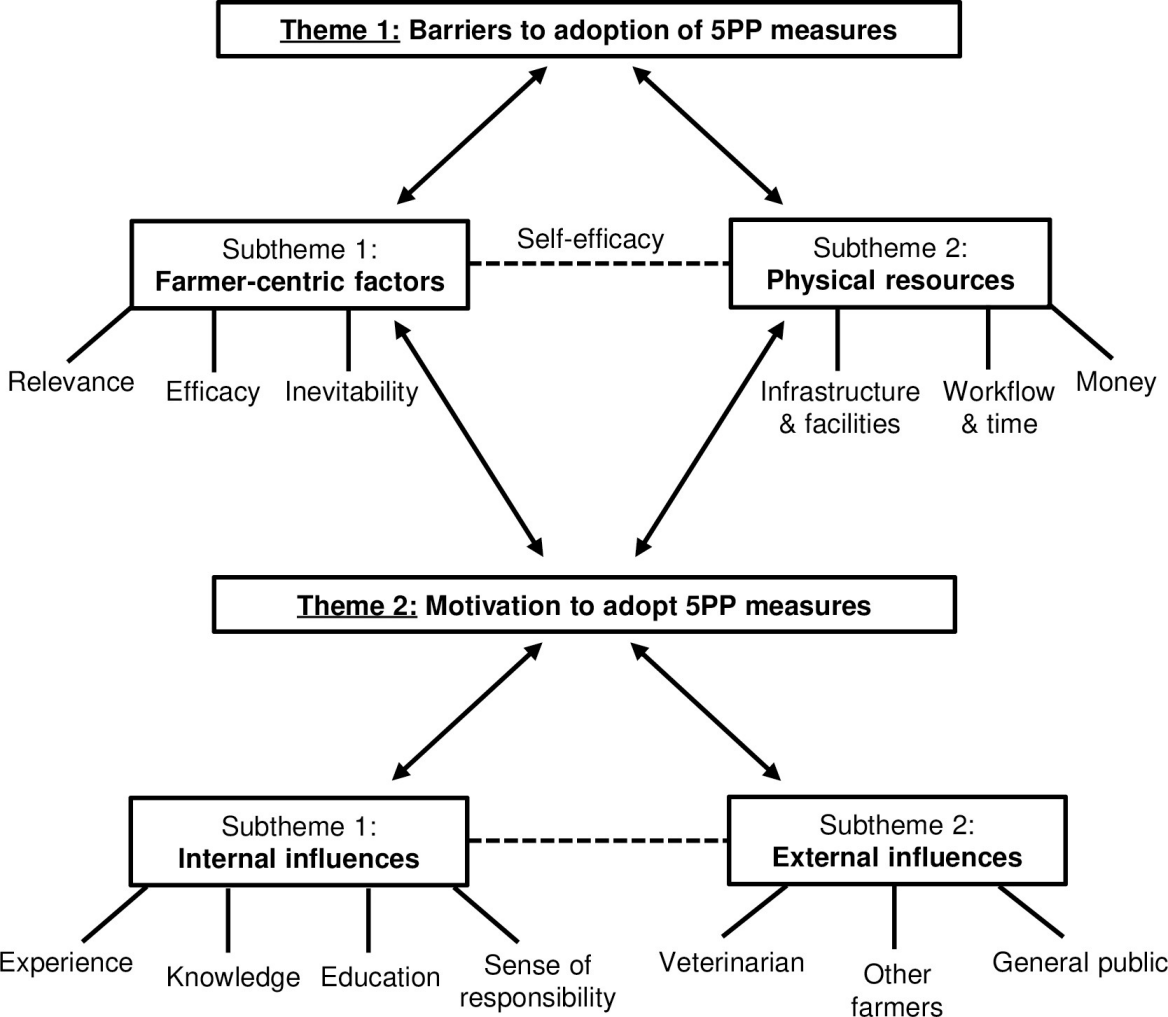
Thematic map demonstrating the inter-relationships between the two overarching themes, subthemes and categories emergent during thematic analysis of 12 interviews.

The results from this exploratory study present the heterogeneity in attitudes towards adopting the 5PP when treating and controlling lameness, from the farmers’ perspective. Two overarching themes, delineated by subthemes, emerged during analysis; (1) Barriers to adoption of 5PP measures and (2) Motivation to adopt 5PP measures. A thematic map to conceptualise themes, subthemes, categories, and their inter-relationships is illustrated in Fig 1.

### Theme 1: Barriers to adoption of 5PP measures

Farmer perceived barriers to implement 5PP measures were consistently evident within the interview data. Barriers presented an obstacle that limited farmers’ ability to carry out 5PP measures outright, or restricted farmers’ capacity to make changes to facilitate their adoption. The described barriers were influenced by factors directly impacting farmer perceptions and attitudes towards the 5PP, these were defined as farmer-centric factors, or individual farm circumstances, defined as physical resources. This notion of individuality is concisely expressed by Farmer No. 8:

*“Because what works in the Five-Point Plan won’t necessarily work everywhere*.*”*

#### Subtheme 1.1: Farmer-centric factors

This subtheme was comprised of three emergent categories defined and described as: perceptions of relevance, perceptions of inevitability and perceptions of efficacy. Farmers’ attitudes, perceptions, beliefs and values emerged as important determinants for the adoption of 5PP measures. Here, plan uptake was dependent on farmers’ perceptions of the relevance of measures to their enterprise, farmer mindset particularly relating to lameness as an endemic disease, and perceived efficacy of measures. Whilst farmer-centric factors were generally seen as barriers, some could also be a motivation for change.

Perceptions of relevance: Whilst some farmers prioritised lameness control using the 5PP, other farmers ranked particular measures as a low priority. In some instances, farmers did not consider lameness prevalence to be sufficiently high to warrant specific control measures, or did not perceive eliminating entry of lameness as important when the flock was already affected by CODD and footrot:

*“We’ve got CODD here*, *we’ve got footrot*, *we’ve got most things*, *so from our point of view*, *it’s a matter of containing and keeping it under control as opposed to keeping it out*, *in the current state*.*”* Farmer No. 4

Furthermore, the absence of factors perceived to influence lameness prevalence was also a reason for why farmers did not implement particular practices:

*“Given our low stocking density with our calcareous soils*, *I’m not too worried about transmission*, *so I don’t need to isolate lame sheep*.*”* Farmer No. 1

In some cases, 5PP measures had previously been used to reduce lameness prevalence to the extent that no future intervention was required:

*“I pulled out one hundred and sixty ewes out of six hundred which were lame*, *and it was anywhere from just a little nod to barely walking*. *I did cull some*, *but got that down to half a dozen by vaccinating and a series of other things… So*, *once we got it down*, *the incidence of lameness going forward was next to nothing*. *We just got on top of it*.*”* Farmer No. 5

Perceptions of inevitability: Farmers considered lameness an inevitable consequence of farming sheep and acknowledged the impact of this endemic disease on personal well-being. Farmers were worried and anxious about lameness, and others felt disheartened as they were unable to eliminate lameness unlike other diseases:

*“No matter how hard you try culling and treating*, *you always end up with some lameness*. *It’s probably the thing that drives me most mental about sheep… everything else you can deal with*, *but lameness is always there*, *or seems to be*.*”* Farmer No. 9

*“We’re constantly worrying about lameness*, *especially CODD and footrot*. *I feel that we can deal with Campylobacter and abortions*. *It’s the things you can’t cure are our concerns*.*”* Farmer No. 4

Perceptions of efficacy: Farmers were critical of 5PP measures and were only motivated to implement measures perceived as efficacious. Farmers considered 5PP measures to be effective when visible reductions in lame sheep and antibiotic treatments were observed following implementation:

*“I can’t really remember what we had before… ten*, *fifteen or twenty per cent* [lameness] *and they would all be hobbling around and then you Footvax and if you saw a lame one it was quite a rarity*. *So*, *it does make a huge*, *huge impact and on antibiotic use as well*. *I dare not stop it really*.*”* Farmer No. 2

Therefore, farmers were discouraged to implement measures, or make on-farm changes, if they did not believe proposed measures were effective in controlling lameness. Some farmers expressed doubt in efficacy following a negative past experience:

*“It* [vaccination] *didn’t touch some of them and we had to cull sort of five percent based on bad feet alone… I wouldn’t vaccinate on a routine basis*, *even if I did have an issue*.*”* Farmer No. 1

However, there were discrepancies between perceived efficacy and actual efficacy, where some farmers were reluctant to cease using outdated practices such as prophylactic use of antibiotics or routine foot trimming, which were perceived to be worthwhile:

*“If I had a scald issue*, *I would use Lincocin powder* [lincomycin] *rather than formalin* [formaldehyde]. *I know it’s a broad antibiotic and I’m running sheep through it that don’t necessarily need to be treated*, *but as a topical treatment*, *I believe it is beneficial*.*”* Farmer No. 10

*“I’m afraid I don’t agree in not trimming feet… we would pare back the horn and then we give them an antibiotic*. *I think it works*.*”* Farmer No. 5

#### Subtheme 1.2: Physical resources

This subtheme was comprised of three emergent categories defined and described as: infrastructure and facilities, workflow and time, and money. Farmers emphasised physical impediments, such as restrictions on facilities, workflow and time, and money, as recognisable barriers to the implementation of 5PP measures. Conversely in some situations, constraints on physical resources were considered a motivation to adoption.

Infrastructure and facilities: Farmers described a number of physical restrictions which impacted their ability to implement lameness control practices successfully and were considered barriers to implementation. For example, shortage of land and building space made quarantining large numbers of bought in stock impractical:

*“We talk about quarantine*, *but again*, *it’s the practicalities around doing things like that*. *I mean*, *if I brought in a handful of rams*, *then yeah*, *I would keep them in a stable*, *that’s not a problem*, *but if you’re buying in numbers*, *then that’s hard when you don’t have space to do it*.*”* Farmer No. 10

Most farmers were critical of their handling facilities which limited their ability to handle sheep regularly. As a result, treatment of lame sheep was delayed:

*“I do see the value in fast treatment*, *so I do try*, *but sometimes*, *if it’s a difficult field where I can’t really corner them well or I don’t have hurdles to make a pen*, *then I have to leave them until I can round them all up*.*”* Farmer No. 9

Farmers also felt that not having a recording system in place was a barrier to recording lameness events to inform later culling decisions:

*“We aren’t very good at culling*, *I must admit*, *as we don’t have a system in place for recording two strikes and you’re out*.*”* Farmer No. 4

Workflow and time: Treating lameness was considered “*hard work”*. Farmers with mixed farms highlighted that their time was spread thinly across many different enterprises, which resulted in delayed treatments:

*“We’re probably not as quick as we ought to be*, *but because we’ve got so many different other things on our farm*, *it’s a case of treating when they’re next handled*.*”* Farmer No. 6

Time of year was also perceived to impact farmers’ ability to implement measures to treat and control lameness. Separating lame ewes with lambs at foot was considered impractical:

*“Well as soon as they haven’t got lambs*, *then we’ll isolate them*. *But while they’re running lambs until weaning*, *then you can’t isolate them as you can’t catch their lambs*.*”* Farmer No. 5

Others highlighted that only a small window of opportunity was available to cull lame ewes, resulting in greater lenience towards lame sheep:

*“There are certain periods of the year when you can’t cull a ewe*, *so when she has just lambed and you’re probably not going to cull a ewe that’s heavily pregnant or half-way through her pregnancy even*, *until she’s finished rearing her lambs*. *So*, *if she’s had a course of treatment in that time or even two courses and is then better and is a young ewe in July*, *are you really going to cull her*? *Probably not*.*”* Farmer No. 4

However, some farmers recognised the impacts of lameness on their time and were motivated to employ preventative control measures:

*“By breeding or culling my problems out*, *I am spending less time treating*.*”* Farmer No. 1

Money: Financial constraints were a fundamental barrier to implementing measures. Vaccination against footrot was considered costly:

*“To go through every ewe in the three thousand to vaccinate it*, *that’s a cost*.*”* Farmer No. 1

Some farmers considered larger flocks to be more profitable and accordingly, were reluctant to cull repeatedly lame ewes until the target flock size was achieved:

*“It’s a numbers game*, *you need a certain amount of sheep to make it profitable*, *so it can get to the stage where you’re ending up saying ‘oh keep her*, *keep her’*.*”* Farmer No. 10

Buying in replacement ewes was considered instrumental to success, irrespective of disease risks:

*“We accept it is a biosecurity weakness*, *but what we gain from having those ewes and the number of lambs sold is what we are looking at… I want to buy in stock*, *so I am willing to take a certain amount of risk*, *because I feel I get more benefit*.*”* Farmer No. 4

However, some farmers recognised the financial costs of lameness, particularly antibiotic treatments, and were motivated to employ control measures in order to reduce subsequent treatment costs:

*“We were using antibiotics like they were going out of fashion*, *and we were ending up using the antibiotics we didn’t want to use*, *they were very expensive and they were the ones you wanted to keep in the cabinet for a just in case job*. *So*, *we decided we were going to start vaccinating*.*”* Farmer No. 8

Others appreciated the indirect costs of lameness on reduced ewe and lamb performance, which motivated them to employ control measures:

*“Lame sheep and especially lame lambs are affected quite a lot by not performing as well*, *and lambing percentages go down because they’re lame*, *moving them takes a lot longer*, *and obviously if the lambs are lame*, *they’ll lose weight*.*”* Farmer No. 6

The benefits of joining flock health clubs were recognised, as enhanced access to veterinary advice exceeded membership costs:

*“I’m emailing her and talking to her* [the veterinarian], *probably every week about something*, *so I’m getting my money’s worth*.*”* Farmer No. 3

However, some farmers were reluctant to seek veterinary input as it was perceived to be costly:

*“I’m not willing to get the veterinarian involved*, *as it’s just an additional cost*.*”* Farmer No. 1

### Theme 2: Motivation to adopt 5PP measures

A number of motivational factors important to the adoption of 5PP measures emerged from interview data. Whilst these factors increased farmers’ willingness to adopt 5PP practices, their readiness to make changes to facilitate implementation and their commitment to sustain implementation, insufficient motivation limited or prevented the implementation of 5PP measures. Motivations were linked to internal influences, or characteristics of the individual farmer, and external drivers such as social norms and relationships. The influence of internal and external motivators is succinctly expressed by Farmer No. 10:

*“I hate to see lame sheep and you just don’t want people to see them*.*”*

#### Subtheme 2.1: Internal influences

This subtheme was comprised of four emergent categories defined and described as: experience, knowledge, education and sense of responsibility. These themes of farmer attributes and characteristics were linked to confidence and motivation to implement 5PP measures. However, some factors demotivated farmers, negatively impacting 5PP adoption.

Experience: As a result of working experience, most farmers were confident in their ability to diagnose lameness and were familiar with lameness risk:

*“Once you’ve been doing it several seasons*, *you know the sheep and you know the ground*.*”* Farmer No. 1

Farmers who considered themselves experienced were reluctant to request veterinary involvement when diagnosing or treating lameness, unless a problem was perceived to be beyond their capabilities:

*“We’re quite experienced… I think we can diagnose as well as they* [the veterinarian] *can diagnose*. *If we had a problem which we felt we couldn’t deal with*, *then we would go straight to them*.*”* Farmer No. 4

However, adverse past experiences of lameness, such as outbreaks, motivated farmers to implement control measures:

*“If we don’t keep on top of it*, *you end up with a problem*.*”* Farmer No. 6

Also, farmers learnt from previous negative experiences:

*“I had a couple of ewes that were lame and treated*, *and I thought ‘oh they’re looking ok*, *they’ll be fine’*, *put them back with the ewes… bad idea*. *Because it was everywhere and it proved how easily it spreads*, *just from a couple of ewes*, *and ever since then*, *until their feet are sound and the infection has gone*, *they don’t go back with the others*. *Because*, *like I say*, *I’ve learnt from my mistake*.*”* Farmer No. 8

Knowledge: Farmers were generally confident of their own knowledge, awareness, and theoretical understanding of the causes and transmission of lameness. Although sufficient knowledge influenced pro-activity and implementation of the 5PP, incorrect knowledge was still an influencing factor. Existent knowledge was predominantly assimilated from intrinsic sources, such as experience, rather than extrinsic sources, such as veterinarians.

Farmers considered ewes to be most susceptible to footrot when housed for lambing and correspondingly, farmers were motivated to reduce disease challenge during this time:

*“They are challenged by being indoors and they’re challenged when their whole immune system is being reduced when they lamb*. *Vaccination coincides with bringing them indoors*, *because that is the period of the year when our sheep are more vulnerable to footrot*.*”* Farmer No. 4

Farmers also recognised the transmission of lameness between ewes and lambs:

*“You see that little line of ewe and two lambs following behind her*, *you see her lame on her front left*, *so will the two lambs behind her and they are treading on that same bacteria*, *you know*, *they are following that same track*, *and it just… it’s a never-ending cycle*.*”* Farmer No. 10

As a result, farmers saw the benefits of culling repeatedly lame sheep to minimise the reservoir of infection and reduce lameness risk:

*“If you get rid of them*, *then that’s how you keep lameness out of the flock*, *because you haven’t got them hanging around*.*”* Farmer No. 5

Farmers considered buying in sheep to be an important biosecurity weakness associated with the transmission of lameness, particularly the introduction of CODD and novel strains of footrot. To mitigate risk, some farmers prioritised purchasing stock from a trusted, reputable source:

*“When we used to come from market*, *we would buy in from several different farmers and it was just a nightmare*. *The fact that we now get all sheep from the same place means that they’ve been exposed to the same… we’re not bringing anything new onto the farm and so yeah*, *there’s less disease transmission*.*”* Farmer No. 6

However, some farmers did not recognise that interdigital dermatitis (scald) and footrot were two clinical presentations of the same disease. As a result, treatment and isolation of ewes with footrot were prioritised over those with scald:

*“So*, *as it’s just mainly scald*, *there’s no point in isolating it*, *as it’s just a chaffing or an irritation when the grass rubs between their cleats*. *It’s just not a priority*. *If we had footrot… I would take time to isolate them*, *mark them up and treat them*, *but it’s just not required for scald*.*”* Farmer No. 12

Furthermore, some farmers were confused, but showed conviction in their knowledge, when comparing the aetiology and treatment of lameness between sheep and cattle. One farmer foot trimmed sheep for the same reasons as for cattle:

*“I appreciate the advice is not to trim*, *but if I see an overgrown foot… I do it with the dairy cows on a daily basis… so we routinely trim*, *and we will trim a lame sheep*.*”* Farmer No. 7

Education: Farmers raised the importance of updating their knowledge of lameness through educational opportunities. One farmer learnt of CODD at a local veterinary-led meeting and was able to implement the acquired knowledge immediately on farm:

*“I would say that it’s* [CODD] *only something we’ve seen in the last couple of years and that’s potentially because we’ve realised what it is… We went to a farm meeting and that’s when we realised*, *‘oh there’s two* [CODD and footrot], *we better have a look at them when we get home’*.*”* Farmer No. 8

However, attending meetings was sometimes a reason for why veterinary input was not required:

*“There’s not many things that we would worry about needing the veterinarian to come out to do or treat as we go to a lot of farm meetings*, *you know Farming Connect stuff and veterinarian stuff*, *we’re kind of up to date and using the right things*.*”* Farmer No. 8

Although meetings weren’t always accessible to farmers:

*“In this part of the world it’s all dairy*, *there’s a lot of dairy meetings at nights*, *but never any sheep meetings whatsoever*, *which would be nice if there were*, *but no one wants to sponsor a sheep meeting where there are no sheep*.*”* Farmer No. 12

Sense of responsibility: Farmers acknowledged their desire and responsibility to conform to high health and welfare standards. Farmers discussed the stigma and shame associated with having lame sheep:

*“I feel quite embarrassed*, *even if it was one out of a field of a hundred sheep*, *I would feel embarrassed and responsible… I hate seeing them lame*. *I feel it reflects badly of me*.*”* Farmer No. 2

As a result, farmers prioritised prompt treatments to limit the impacts on sheep health and welfare:

*“We don’t leave them hanging about at all*, *if you’ve got an issue then it needs to be dealt with quickly otherwise you’ll end up with a worse situation*. *And it’s not fair on the sheep*.*”* Farmer No. 12

#### Subtheme 2.2: External influences

This subtheme was comprised of three emergent categories defined and described as: veterinarians, other farmers and general public. Farmers were influenced by social interactions with different stakeholders, where veterinarians, other farmers, and the general public were identified as key influences within farmers’ social connections and were considered to be motivators for farmers to act against lameness.

Veterinarians: All farmers valued veterinarians with sheep experience, but they were considered a rarity. Farmers considered themselves fortunate to have access to a veterinarian with sheep interest:

*“Our veterinarian is good with the sheep*, *she is really keen*, *but the rest of them would sack off the sheep and just focus on the dairy if they could*.*”* Farmer No. 12

These veterinarians provided proactive advice on lameness and worked alongside farmers, which was favoured by farmers:

*“So*, *they’re very much about working with us rather than firefighting*.*”* Farmer No. 6

However, this was not the case for all farmers. Some farmers described a poor working relationship with their veterinarian, which resulted in their veterinarian being used as a drugs supplier:

*“Our veterinarian is not a good large animal veterinarian*. *So*, *we don’t have a great deal of help from him* [the veterinarian], *it’s usually us telling him what we’ve got and asking for the drugs*, *rather than him being proactive*. *We should really change*.*”* Farmer No. 5

Other farmers: Farmers were more motivated to implement measures when reputable, trusted farmers were known to use them, than from a veterinarian’s recommendation. This was stressed by one farmer in the case of vaccination:

*“A veterinarian could say it and they’re a bit like ‘oh*, *whatever’… but when other farmers have used it*, *that of course makes us more inclined to use it*, *‘oh so and so at market have used it*, *so it must be a good thing’*. *That has a huge influence I would say*.*”* Farmer No. 2

However, farmers were quick to criticise the lameness prevalence on neighbouring farms:

*“I do get very cross with farmers*, *I just don’t understand how they all have foot problems*. *We’ve got two neighbours and when they’re moving their sheep on the road*, *a huge percentage can’t walk and it’s an absolute disgrace*.*”* Farmer No. 5

Although comparing to other farmers’ experiences of lameness instilled farmers with some confidence:

*“You hear some horror stories from other people about lameness explosions in the lambing shed*, *but we must be doing something right*, *as it isn’t an issue for us*.*”* Farmer No. 12

General public: Farmers perceived the general public to have a limited understanding of sheep lameness. Farmers were concerned about negativity, and correspondingly, made efforts to conceal lame sheep in *“hospital paddocks”* away from roads and footpaths:

*“People get verrucas*, *people get sore toes… human’s get ill*, *we have hospitals*, *and this is our hospital… that is something that people need to realise*, *animals do go lame*, *it is nothing that we’re doing wrong*, *per say*, *and it’s just one of those things that we are dealing with and helping*.*”* Farmer No. 10

In light of negative public perception and fractured public relationships, farmers wanted to be seen to be pro-active:

*“I do tend to act immediately if somebody says something*, *because you’ve got to*, *haven’t you*? *Otherwise*, *it’ll go downhill*. *But there’s lots of people out there looking*.*”* Farmer No. 3

Farmers believed that the general public were concerned about antibiotic usage in the sheep industry, thus favoured control measures that reduced reliance on antibiotic treatments:

*“I would rather vaccinate* [Footvax®] *than fill them with antibiotics*. *Because that’s not doing our public perception any good*.*”* Farmer No. 8

## Discussion

This is the first qualitative study to explore the factors affecting farmers’ adoption of the 5PP, the national program to reduce and control lameness in UK flocks. It is evident that farmers’ attitudes and subsequent adoption of the 5PP are influenced by a complex interplay between intrinsic factors, social dynamics and influences, and physical resources, presenting as both barriers and motivation.

### Barriers to adoption of 5PP measures

#### Intrinsic factors

Negative farmer mindset, such as expressing concern, worry and anxiety over the inevitability or unpredictability of lameness, was a cognitive barrier to 5PP adoption. Some farmers voiced negative emotions and dominant feelings of hopelessness towards the control of lameness, which have been associated with increased lameness risk [23]. Furthermore, farmers perceived lameness practically more difficult to control than other diseases, mirroring views from dairy farmers [21]. As a result, some farmers in our study lacked enthusiasm to implement new 5PP measures, experienced fatigue in continuing to implement existing measures, or perceived bleak benefits following implementation.

Farmers perceived some 5PP measures to be unnecessary, not applicable or irrelevant to their specific circumstances, which hindered their implementation on farm. These perceptions were reasoned by farmers’ attitudes towards lameness risk and current circumstances. As a result, some farmers failed to acknowledge the need to implement these measures, or reduced their priority, similar to Johne’s disease non-compliance on dairy farms [36]. Increasing farmers’ awareness of the scientific reasoning and benefits behind the importance of measures could increase 5PP compliance.

Perceptions of poor efficacy of individual 5PP measures was an important barrier to implementation. It is widely recognised that dairy farmers are reluctant to implement disease control measures considered to be ineffective [11, 37, 38]. In our study, negative perceptions of practices were informed by adverse past experiences; farmers who previously reported negligible impacts of vaccination on lameness were less likely to choose to vaccinate in the future, analogous to dairy farmers [39]. In some cases, farmers were quick to cease Footvax® vaccination if visible reductions on lameness were not observed within a short time frame. Similarly, farmers have been reported to quickly discontinue practices perceived to fail, despite the investment in time when deciding to implement them [31]. Our findings are of particular concern considering only long-term (> 5 years) implementation of Footvax® vaccination is associated with significantly reduced lameness prevalence [5]. Future message framing needs to increase farmer acceptance that reducing lameness is a long-term strategy, requiring sustained commitment.

We document that farmers’ personal beliefs hindered decisions to implement 5PP practices. Farmers were reliant on their own beliefs to guide decision-making, reflective of tacit knowledge derived from working experience passed down through generations. In some cases, poor perceptions of 5PP efficacy were informed by preconceived ideas or misconceptions, without understanding the scientific reasoning informing recommendations. Personal judgement, informed by beliefs and not scientific advice, was previously identified as a barrier to adopting best-practice recommendations when treating lame sheep [26]. Reliance on own judgement has also been identified as a barrier to sheep farmers adopting sustainable parasite control [13]. Failure to acquire unbiased knowledge, whilst relying on own views, presents an important barrier to 5PP implementation. Increasing the perceived credibility and trust of external sources documenting 5PP efficacy could prove worthwhile in reducing ambiguity and motivating farmers to make long-term changes to lameness management.

As a result of expertise built through years of farming experience, the majority of farmers in our study considered themselves to be experts in sheep farming, reflective of previous research [40]. In some cases, experience had a negative influence on adopting 5PP measures. More experienced farmers are less likely to employ sustainable parasite control practices [13] and less likely to seek external advice proactively [31]. Furthermore, embedded farming experience has been reported to supersede knowledge acquired from college education when treating lame sheep using best-practice recommendations [27]. Some farmers in our study did not perceive themselves having a deficit in knowledge, which led to inconsistencies in treatment priorities, as previously shown by [26]. Our findings suggest there is still scope for farmer-learning and education, but this could be challenging if not all farmers are able to recognise knowledge deficiencies [41]. Engaging all farmers in knowledge exchange, through participatory or personal communication, could address this shortfall in knowledge acquisition. Otherwise, inadvertently targeting already proactive farmers is unlikely to increase the rate of 5PP adoption.

In some cases, farmers were knowledgeable of the negative impacts of foot trimming or whole-flock treatments, yet chose to continue with these ill-advised measures as they were considered effective. This is an example of cognitive dissonance [42], where people change their beliefs to match their behaviour, even if they know that the behaviour is sub-optimal. Cognitive dissonance was identified as an explanation for why farmers continued to use footbathing [43]. In order to change farmers’ behaviour accordingly to reflect their knowledge, educational dialogue should disincentivise farmers adopting risky behaviours by convincing them of the benefits of ceasing these measures on quantifiable attributes, such as time and money.

#### Social dynamics and influences

In our study, some farmers perceived veterinarians to be disinterested in providing proactive advice on lameness control, which lead to veterinarians being excluded from strategic decisions. Furthermore, some farmers perceived veterinarians to lack specialist sheep experience above their own unique understanding of farming sheep, which has been previously identified as a hurdle to farmer-veterinarian engagement [40]. As a result, these farmers considered veterinarians to provide little additional value, echoing previous findings [13], and had no desire to improve professional relationships. Poor farmer-veterinarian communication limits the veterinarians’ ability to facilitate behavioural change towards biosecurity practices on sheep and cattle farms [44], reduces dairy farmers’ preparedness to adopt veterinary advice [45], leading to suboptimal management and reduced success rates [16]. As a result, some farmers in our study viewed veterinarians as medicine suppliers or offering a ‘firefighter’ service, parallel to findings 10 years ago [40]. This is consistent with findings suggesting that veterinarians too consider themselves to offer an emergency service, rather than consultancy work to prevent disease [46].

Veterinarians were not often seen as the main source of information for sheep farmers in our study, in direct contrast to dairy farmers [47, 48]. Instead, farmers were more likely to approach other farmers for information in the first instance, rather than the veterinarian, similar to dairy farmers seeking advice on digital dermatitis treatments [37]. Our findings suggest that reluctance to consult the veterinarian was a result of considering veterinary advice expensive, and perceiving first-hand farmer knowledge more accessible and valuable. However, reliance on information from other farmers presents a barrier to obtaining unbiased and objective information [30].

#### Physical resources

We report that inadequate or absent physical resources limited farmers’ beliefs in self-efficacy, or perceived ability and confidence, representing an important barrier to implementing the 5PP. This mirrors findings from other farming enterprises [32, 49]. We identify that ease of implementation was a criterion for which farmers used to assess the compatibility, feasibility and practicality of lameness control measures. Limited infrastructure and facilities, sometimes as a result of reluctant investment, impacted farmers’ confidence and beliefs in self-efficacy, hampering their motivation to carry out the measure. This ranged from lack of building space or land, through to handling facilities and flock management technology. This is consistent with reports suggesting dairy farmers justify lack of lameness control with lack of handling facilities [17]. Therein lies scope to increase farmers’ perceived ability and confidence to effectively implement the 5PP, in order to drive uptake.

Unsurprisingly, financial restrictions represented an important barrier to 5PP implementation. Immediate costs of implementing 5PP practices, such as vaccine costs, were unaffordable for many farmers compared to other on-going costs, similar to dairy farmers [39]. Farmers considered some measures as prohibitively costly, such as culling lame sheep perceived as otherwise productive, and were blind to the long-term, holistic benefits. Likewise, dairy farmers have also been reported to focus on the quick economic benefit from lameness control measures, which often hindered the adoption of new measures perceived as expensive [50]. Equally, anticipated financial investment in facilities was also a barrier to improving inadequate infrastructure and adoption of 5PP measures, especially when costs were not justified by perceived benefits. Raising awareness and recognition of the cost-benefits and cost-effectiveness of 5PP measures could increase uptake of measures.

Veterinary input was also considered expensive, as farmers failed to recognise the difference between ‘investment’ and ‘cost’. These negative cost implications reflected the lack of additional value in which veterinarians were perceived to provide. Previous reports suggest that sheep farmers disbelieve veterinarians can play a major role in flock health management, and as a result, considered their time costly [40]. Similarly, dairy farmers have been reported to call out the veterinarian based on a cost-benefit calculation [51]. Therefore, it is imperative that farmers are increasingly aware of the distinction between ‘positive veterinarian spend’ (pro-active) and ‘negative veterinarian spend’ (re-active), as previously framed [52].

Farmers were also less motivated to implement 5PP measures perceived to cause an inconvenience, break a habit or change farming routine, due to excessive workload, lack of labour and logistical issues. Many farmers highlighted the hassle or time anticipated when implementing practices to control lameness, which diminished their motivation to make changes to routine. Perceptions of inconvenience or disruption to farm routine were also enough to disincentivise dairy farmers to implement control measures [18, 20]. In our study, 5PP measures were often rejected outright by farmers, such as isolating lame ewes, vaccinating against footrot or implementing avoidance measures. The balancing act between treating lameness and other day-to-day farming tasks was an important factor limiting the provision of prompt treatments, as stated by dairy farmers [19]. It is likely that measures perceived to be inconvenient or time-consuming were considered unfavourable as little benefits of change were expected. Further efforts should increase farmers’ recognition of the wider benefits of employing lameness control measures, such as the reduction of workload associated with treating lame sheep.

### Motivation to adopt 5PP measures

#### Intrinsic factors

Farmers were motivated to adopt the 5PP when they were knowledgeable of perceived risks of lameness susceptibility and impact. Knowledge of lameness cause and transmission were partly influenced by experience. Previous experience of disease shapes farmers’ perceptions of disease risk and resultant biosecurity compliance [31, 53]. In our study, a severe outbreak of lameness heightened farmers’ awareness and perception of lameness, acting as a trigger event to implement proactive lameness control. Significant trigger events, such as bovine tuberculosis outbreaks, have been shown to initiate farmer behaviour change [54]. In some cases, farmers in our study were hesitant to drop aspects of the 5PP, such as vaccination, from routine flock management as was considered an insurance policy, mirroring veterinarians’ perceptions of vaccine use on dairy farms [39].

Farmers were particularly driven to implement practices considered to make significant reductions to lameness prevalence. This was, in part, due to the pragmatic nature of farmers. Farmers made informed decisions to carry out 5PP practices only when they were considered logical or aligned with their current experience, knowledge and awareness of lameness, supporting other studies [31, 30]. In our study, farmers with a sound knowledge of footrot aetiology and transmission recognised lameness as a multi-factorial disease requiring a comprehensive control strategy; these farmers were motivated to employ all aspects of the 5PP. Reduced risk of lameness has previously been associated with farmers’ understanding of the importance of active lameness control [23]. Raising farmers’ sensitivity of lameness risk perception, through greater knowledge and awareness of lameness, could increase uptake of treatment and control strategies, as previously recommended [17].

Emotional impacts of past lameness outbreaks were important motivators to implementing control measures, similar to the emotional effects of bovine tuberculosis on dairy farmers [54]. Lameness had a detrimental effect on farmers’ morale, which in some cases provided motivation to implement proactive lameness control to safeguard against emotional hardship. This reflects findings suggesting dairy farmers vaccinate against bluetongue virus to avoid perceived emotional confrontation with diseased cattle in their herd [55]. Furthermore, farmers in our study were driven to purchase sheep from trusted sources, as the transmission of novel lameness types, particularly CODD, was a major concern for farmers with disease-free status.

Sense of job satisfaction and responsibility were important intangible factors which had positive impacts on self-esteem and subsequent decision-making. Cultural capital, or sense of pride and esteem associated with particular actions, has been widely attributed to decisions to treat lame sheep [26] and disease control in dairy farms [7, 8]. In our study, farmers considered the timely treatment of sheep and the adoption of strategies to control lameness as a moral obligation, supporting other findings [26].

#### Social dynamics and influences

Farmers were aligned to others within the farming community. Other farmers were positive reference groups and what they think, do and say were instrumental to farmer attitudes in our study. Farmers aspired to be portrayed as a ‘good farmer’ by the farming community, and as such, were motivated to implement 5PP measures to reduce lameness. Similarly, dairy farmers had clear perceptions of the ‘good farmer’ and strove to be recognised as such by other farmers [9]. As a result, farmers in our study were highly critical and frustrated of other farmers’ lack of commitment and poor attempts to control lameness, similar to dairy farmers [56].

As a result of the value placed on relationships within the farming community, farmers’ perceptions of self-efficacy, and the effectiveness of control measures were found to be constructed from other farmers’ experiences of 5PP implementation. Social networks and peer-to-peer learning are important in knowledge transfer and in highlighting the success of other farmers in performing target behaviours [15, 57, 58]. In our study, awareness of successful 5PP implementation on other farms reduced farmers’ doubts and increased farmers’ confidence, which motivated them to employ 5PP measures. Although farmers appreciated the importance of lameness control measures, farmers needed to ‘buy-into’ the concept via peer-to-peer learning, from a ground-up approach. Promoting the successes of other farmers in implementing the 5PP, through demonstration farms or case-studies, could provide an opportunity to increase engagement with sheep farmers by establishing 5PP measures as normative behaviours. Furthermore, utilising ‘peer champions’ could also help discourage use of ill-advised measures, such as foot trimming. Directing efforts to develop tacit knowledge of evidence-based practice could encourage positive behavioural change [27].

Flock health clubs were also an environment considered valuable in knowledge transfer and communication between farmers and veterinarians. These clubs provided ample opportunity for building farmer-veterinarian relationships, which resulted in more opportunities for veterinarians to work proactively with farmers. This is particularly important considering temporal changes in circumstances, such as money and time, requires continual assessment [56], opening an opportunity for veterinarians to support farmers in light of changing priorities. Furthermore, by building trusting relationships, veterinarians can align with farmers’ short- and long-term motivational drivers to help guide the successful delivery of advice [59].

Perceived public disapproval of lame sheep and concerns over antibiotic usage were important motivators to reduce and control lameness by adopting the 5PP. This is unsurprising considering the increasing public concern for farmed livestock [60]. Farmers were highly motivated to maintain a good public image and to reduce the reputational costs associated with lame sheep. This is consistent with findings reporting dairy farmers’ desire to maintain a good public image to be a strong external driver for implementing lameness control practices [22]. Similarly, fear of public outcry was influential to implementation of zoonotic disease control practices by cattle farmers [32].

Our findings suggest that farmers were motivated to employ the lameness 5PP to minimise lameness and antibiotic usage in sheep in an attempt to live up to the expectations of consumers and society, as a social licence to farm. Social licence is defined as “the latitude that society allows to its citizens to exploit resources for their private purposes” [61]. Public attitudes towards sheep welfare, and farmers’ perceptions of these attitudes, influence the way in which farmers act, where demonstration of good animal welfare has and will continue to play a primary role in the social licence to farm [62]. We recommend that future lameness control campaigns should focus on maximising sheep health and welfare to benefit consumer relationships, which for the foreseeable future, will demand improvements to the way in which animals are farmed.

#### Physical resources

For some farmers, the economic burden of lame sheep was an important motivator to adopting the 5PP to reduce the monetary costs of lameness. Specifically, antibiotic costs were a driver to implementing control measures to reduce lameness and reliance on treatments. Awareness of financial losses from disease on the value of control practices and subsequent implementation of preventive strategies, has been widely documented [10, 11, 63]. Our study indicated that farmers who recognised the wider, insidious losses associated with lameness, such as reduced flock profitability, were more motivated to employ 5PP practices. Furthermore, these farmers could justify the costs of investing in facilities; significant logistical barriers and practicalities were more likely to be overcome when farmers recognised the cost-benefits of implementing measures [18, 31]. Whilst this awareness is reassuring, it raises the question of why some farmers do not recognise the costs of lameness. Some farmers may lack confidence in published figures, or consider them sensationalist, whilst some may feel they are irrelevant to their flock, opening an opportunity to reduce miscommunication.

Farmers saw their time as a valuable, finite resource. Although lack of time was presented as a barrier for some farmers, others considered it a motivator to implementing lameness control measures. The wider costs of lameness on time and labour have previously been reported as motivations for dairy farmers to reduce lameness [22]. Some farmers were motivated to vaccinate against footrot to avoid the time and labour costs associated with treating lame sheep, especially considering the physical difficulties in catching and handling sheep. Farmers recognised that sheep farming is a labour-intensive enterprise and minimising time spent treating lame sheep using preventative measures would free up time to invest in other areas of management seen as beneficial to sheep health. This is an important aspect which could be promoted to engage farmers in adopting the 5PP.

### Study restrictions

We used established methodological approaches whilst conducting our study, which provides confidence in the findings reported. Farmer participants were at ease and able to comfortably answer questions, regardless of interview modality. As a result, data obtained from face-to-face and telephone interviews were equally data-rich and robust with respect to breadth and depth. Whilst face-to-face interviews are considered the gold-standard, telephone interviews are increasingly popular due to their increased accessibility, anonymity and convenience, facilitating acquisition of detailed, rich data [64]. Furthermore, face-to-face and telephone interviews are reported to provide comparable data [65, 66], and have been successfully utilised concurrently in qualitative data collection from farmers [67, 68].

Results from our study are based on a purposive sample of sheep farmers from England and Wales, and as such, we cannot claim to describe a representative sample of sheep farmers as the perspectives of farmers in this study may differ from other farmers. However, use of purposive sampling meant that a diversity of farmers and enterprises were strategically included in this study.

## Conclusions

As a voluntary strategy, uptake and impact of the 5PP at flock and national level is dependent upon farmers’ opinions, attitudes and decision-making. This study provides valuable insight into sheep farmers’ perceptions of the barriers and motivators influencing the adoption of the lameness 5PP, providing broader implications for strategies to control infectious lameness worldwide. We document the heterogeneity in farmers’ perceptions towards the 5PP, and the complexities influencing whether or not the 5PP is employed on farm.

Although lack of physical resources, such as money and facilities, were considered to impede adoption of the 5PP, non-monetary intrinsic factors were also significant barriers. For the most part, barriers identified in our study could be overcome by changing farmers’ perceptions, mindset and confidence in order to facilitate implementation of practices. Exploiting motivators identified in this study could also help drive positive changes to mindset and confidence. Increasing peer-to-peer learning and promoting positive testimonies or experiences could further increase farmer engagement, confidence and long-term implementation of the 5PP. Emphasising the tangible benefits of measures in constructs important to farmers, such as money and time, could also increase motivation, particularly when highlighting the wider, holistic benefits of lameness control. This could also include benefits of lameness control on the reputational costs associated with lame sheep, raising the position of farmers within society and empowering farmers’ social licence to farm. There also lies an important opportunity for the 5PP to be drawn up in flock health plans in collaboration with the veterinarian to help farmers work towards feasible solutions and targets, and break down barriers to 5PP adoption. Here, we recommend that future campaigns should promote the 5PP as a framework in which should be tailored to individual farms, based on their individual circumstances.

## Supporting information

S1 File Semi-structured interview schedule for participating farmers. (PDF)
