# Supplementary material for: Sheep farmers’ attitudes towards lameness control: Qualitative exploration of factors affecting adoption of the lameness Five-Point Plan
Source: PLoS One. 2021 Feb 9;16(2):e0246798. doi: 10.1371/journal.pone.0246798 (PMC7872274; doi:10.1371/journal.pone.0246798)
Supplement: S1 File — (PDF) [file pone.0246798.s001.pdf]

S1 File

S1. Semi-structured interview schedule for participating farmers

Would you like to tell me about yourself, your farm and your flock?

- *Can you tell me more about where your lambs are sold?*
- *What other livestock do you keep?*
- *How many other people are involved with the flock? Can you describe their roles?*
- *What are your thoughts on the future of your flock? Do you have any future plans?*

From your experience, what have been your three greatest flock health concerns over the past year?

- *Can you elaborate on which were the most difficult to control?*
- *Can you elaborate on which were the costliest?*

Could you tell be about the lameness in your flock?

- *Tell me more about your current lameness prevalence*
- *How often is your flock affected by FR?*
- *How often is your flock affected by scald (interdigital dermatitis)?*
- *How often is your flock affected by CODD?*
- *What are your thoughts on calling the vet for advice concerning lameness?*
- *What are your thoughts on the public awareness of lameness?*

Are you aware of the lameness Five-Point Plan?

How have you adopted the lameness Five-Point Plan?

- *Can you tell me more about which elements of the Five-Point Plan you implement on farm?*
- *Can you elaborate on why you implement these points?*
- *In your opinion, which points do you feel are most challenging to implement?*
- *In your opinion, which points do you feel you are most able to implement?*
- *In your experience, have you sought advice from your vet when implementing the Five-Point Plan?*

How do you avoid the transmission of lameness across your flock?

- *Can you describe what measures are implemented at lambing or housing?*
- *Can you describe what measures are implemented when gathering sheep?*

How do you treat lame sheep with footrot?

- *Tell me more about how you would treat a lame sheep with footrot*
- *Can you elaborate on why you treat lame sheep in this way?*
- *In your experience, how quickly are lame sheep treated?*
- *Have you sought advice from your vet when treating lame sheep?*
- *What types of situation might you seek advice from the vet?*

What are your attitudes towards repeatedly lame sheep?

- *Do you cull repeatedly lame sheep?*
- *Can you elaborate on why you do/ do not do this?*
- *In your experience, after how many episodes of lameness are sheep culled?*
- *In your experience, would you keep their lambs as replacements?*
- *In your opinion, would you cull a sheep with bad hoof shape?*
- *Do you think there is a genetic association with lameness?*
- *Can you elaborate on where lameness lies against other culling priorities?*
- *Can you tell me more about how you monitor and record lameness?*

What happens when you buy in sheep?

- *Do you routinely quarantine bought in sheep?*
- *Can you elaborate on why you do/ do not do this?*
- *How long are they isolated for?*
- *Can you describe how and where the sheep are isolated?*

Do you vaccinate against footrot?

[If yes]:

- *Can you describe your vaccination protocol?*
- *Can you elaborate on why you vaccinate?*
- *In your opinion, what are the advantages of vaccinating?*
- *In your opinion, what are the disadvantages of vaccinating?*
- *In your experience, has vaccination had an impact on your antibiotic usage?*
- *In your experience, has vaccination had an impact on your lameness rates?*

[If no]:

- *Can you elaborate on why you do not vaccinate?*
- *In your opinion, when would you consider using vaccination?*
